# Nickel Ions Selectively Inhibit Lipopolysaccharide-Induced Interleukin-6 Production by Decreasing Its mRNA Stability

**DOI:** 10.1371/journal.pone.0119428

**Published:** 2015-03-05

**Authors:** Sanki Asakawa, Yu Kishimoto, Takayuki Takano, Kiyuki Okita, Shiho Takakuwa, Taiki Sato, Masahiro Hiratsuka, Osamu Takeuchi, Noriyasu Hirasawa

**Affiliations:** 1 Laboratory of Pharmacotherapy of Life-Style Related Diseases, Graduate School of Pharmaceutical Sciences, Tohoku University, Sendai, Miyagi, Japan; 2 Laboratory of Infection and Prevention, Institute for Virus Research, Kyoto University, Shogoin Kawara-cho, Sakyo-ku, Kyoto, Japan; University of Leuven, Rega Institute, BELGIUM

## Abstract

Nickel (Ni) ions easily elute from many alloys and elicit inflammation and allergies. Previous studies have shown that infections due to the implantation of medical devices cause inflammation and enhance the elution of Ni ions (Ni^2+^). However, cross-talk between infection- and Ni^2+^-induced signaling pathways has not yet been elucidated in detail. In the present study, we investigated the effects of Ni^2+^ on the lipopolysaccharide (LPS)-induced production of cytokines in a LPS-induced air pouch-type inflammation model in BALB/c mice and the murine macrophage cell line RAW264. We demonstrated that Ni^2+^ inhibited the LPS-induced production of interleukin (IL)-6, but not that of tumor necrosis factor (TNF)-α both *in vivo* and *in vitro*. This inhibitory effect was also observed with cobalt ion (Co^2+^), but not with chloride ion (Cl^-^), zinc ion (Zn^2+^), or palladium ion (Pd^2+^), and was highly selective to the production of IL-6. Ni^2+^ did not inhibit the activation of ERK1/2, p38 MAPK, or JNK. Although Ni^2+^ decreased IL-6 mRNA levels, it failed to inhibit the LPS-induced activation of the IL-6 promoter. An experiment using actinomycin D, a transcription inhibitor, revealed that Ni^2+^ decreased the stability of IL-6 mRNA. Moreover, Ni^2+^ inhibited the LPS-induced expression of Arid5a, but not regnase-1. These results demonstrated that Ni^2+^ may have selectively inhibited the LPS-induced production of IL-6 by decreasing the Arid5a-dependent stabilization of IL-6 mRNA.

## Introduction

Nickel ion (Ni^2+^) is the most frequently detected contact allergen among patients with allergic dermatitis. The prevalence of metal allergies is very high worldwide, with approximately 65 million people being estimated to be sensitized to Ni^2+^ in Europe alone [1]. Nickel (Ni) allergies are easily and frequently induced because many alloys such as biomedical devices and accessories contain Ni and Ni^2+^ easily elutes from these materials. A previous study reported that Ni^2+^ eluted from coins, mobile phones, earrings, and scissors, and caused contact dermatitis [2].

Ni^2+^ and Ni particles have the potential to elicit inflammation, allergies, and cancer [3, 4]. Ni^2+^ can bind to soluble proteins or peptides/proteins on antigen-presenting cells [5]. Antigen-presenting cells recognize the Ni^2+^-protein complex as an antigen and activate naïve T cells [6], which subsequently differentiate to Ni^2+^-specific interferon γ (IFN-γ)-producing CD4^+^ and CD8^+^ effector T cells [7] and cause Ni allergies. Ni^2+^ and water-insoluble Ni particles can also be taken into cells by ion channels and phagocytosis, respectively [4], and activate various signaling pathways that enhance allergic reactions as the second signal [8].

The elution of metal ions from medical devices was previously shown to cause inflammatory reactions such as eczema and redness [9]. The elution of Ni^2+^ from materials is simultaneously regulated by inflammation. The implantation of medical devices such as prosthetic joints causes infections and inflammation [10]. We demonstrated that the elution of Ni^2+^ from Ni wires was enhanced by a treatment with lipopolysaccharide (LPS) [11]. Furthermore, LPS was previously shown to promote sensitization to Ni^2+^ [12] and augmented allergic responses induced by Ni^2+^ in Ni allergy model mice [13]. Thus, LPS-induced signals may also be the second signal for Ni allergies. However, cross-talk between LPS- and Ni^2+^-induced signaling pathway has not been examined in detail.

Interleukin (IL)-6 is an inflammatory cytokine that was initially characterized as a growth factor of B cells and inducer of immunoglobulin production by B cells [14]. IL-6 is produced by monocytes and macrophages in the early phase of infection-induced inflammation to provide protection against pathogens [15]. It has also been shown to induce the release of chemokines in smooth-muscle cells as well as the recruitment of immune cells [16]. When stimulated with IL-6, hepatocytes secrete acute-phase proteins such as C-reactive protein and serum amyloid A [17]. IL-6 also plays an important role in the progression of autoimmune diseases including rheumatoid arthritis and Castleman disease [18].

IL-6 is known to affect the immune balance. It acts on T cells and induces Th2 responses by inducing the expression of transcription factors that produce Th2 cytokines [19] and also blocking IFN-γ signaling that promotes Th1 responses [20]. IL-6 in the presence of transforming growth factor-β (TGF-β) can also induce the expression of RORγt and promote Th17 differentiation [21, 22].

The expression of IL-6 is regulated at the transcriptional level by transcription factors such as NF-κB [23]. Ni^2+^ was also shown to induce the production of IL-6 by activating hypoxia-inducible factor-1α (HIF-1α) in addition to NF-κB [4] in human endothelial cells. Furthermore, IL-6 was also regulated at the post-transcriptional level by regnase-1 [24] and Arid5a [25].

In the present study, we analyzed the effects of Ni^2+^ on the LPS-induced production of cytokines, such as IL-6 and tumor necrosis factor (TNF)-α, both *in vivo* and *in vitro* in an attempt to better understand cross-talk between LPS and Ni^2+^-induced signaling. Unexpectedly, Ni^2+^ only inhibited the production of IL-6 among the LPS-induced cytokines/enzymes examined. The underlying molecular mechanisms were examined at the transcriptional and post-transcriptional levels.

## Materials and Methods

### Materials

LPS derived from *Escherichia coli* O111, nickel chloride (NiCl_2_), cobalt chloride (CoCl_2_), zinc chloride (ZnCl_2_), palladium chloride (PdCl_2_), nickel sulfate (NiSO_4_), and actinomycin D (AcD) were purchased from Wako Pure Chemical Ind. (Osaka, Japan). Poly(I:C) and zymosan A from *Saccharomyces cerevisiae* were purchased from Tocris Cookson (Bristol, UK) and Sigma-Aldrich (Milan, Italy), respectively. The pRL-TK renilla luciferase vector (control) was purchased from Promega (Madison, WI) and pGL3-IL-6 promoter (-1232 to +39) [26] was supplied by Dr. T. Kishimoto, Osaka University, Japan and Dr. A. Kimura, Keio University, Japan.

### LPS-induced air pouch-type inflammation in mice

Male BALB/c mice (specific pathogen-free; SLC, Shizuoka, Japan) were treated in accordance with procedures approved by the Animal Ethics Committee of the Graduate School of Pharmaceutical Sciences (Tohoku University, Sendai, Japan).

The induction of LPS-induced air pouch-type inflammation and analysis of inflammatory responses were performed according to a method reported previously [27] with minor modifications. Briefly, mice were injected subcutaneously with 4 ml of air on the dorsum and, 6 days later, 2 ml of air was added to the pouch. The next day, NiCl_2_ (30 or 300 μM) and LPS (10 ng/ml) were dissolved in a sterile solution of 2% (w/v) sodium carboxymethylcellulose (Cellogen F3H; Daiichi Kogyo, Niigata, Japan) in saline supplemented with 0.1 mg/ml penicillin G potassium and 0.1 mg/ml streptomycin sulfate, and 2 ml of the solution was injected into the air pouch. Mice were sacrificed 8 hours after the injection and the pouch fluid was collected and weighed. The pouch fluid was diluted with saline, the number of cells was measured using a hemocytometer, and the concentrations of IL-6, TNF-α, IL-1β, and monocyte chemoattractant protein-1 (MCP-1) in the supernatant of the diluted fluid were measured using an ELISA following the manufacturer’s protocol. IL-6 and TNF-α ELISA reagent kits were purchased from eBioscience (San Diego, CA) and IL-1β and MCP-1 ELISA reagent kits were from R&D Systems (Minneapolis, MN).

### Treatments with stimulants and inhibitors

LPS, NiCl_2_, CoCl_2_, ZnCl_2_, PdCl_2_, NiSO_4_, poly(I:C), and zymosan A were dissolved in water and AcD was dissolved in ethanol and diluted with Eagle’s minimal essential medium (Nissui, Tokyo, Japan). The final concentration of ethanol was adjusted to 0.1% (v/v). All stimulants are soluble at the concentrations used in this study.

### Cell culture

The murine macrophage cell line RAW264 (Riken, RCB0535) was used in the present study. Cells were cultured at 37°C under a humidified atmosphere of 5% CO_2_–95% air in Eagle’s minimal essential medium (Nissui) containing kanamycin (60 μg/ml) and 10% (v/v) heat-inactivated fetal bovine serum (FBS, Biowest, Miami, FL). Cells were detached and seeded in each well of a multi-well plate (Becton, Dickinson and Company, Franklin Lakes, NJ) as described below.

### ELISA

RAW264 cells (2.5 × 10^4^ cells/well) were seeded onto 96-well plates, and stimulated the next day as described above. After being incubated for the indicated times, IL-6 and TNF-α in the medium were assayed using an ELISA kit (eBioscience) following the manufacturer’s protocol.

### MTT assay

RAW264 cells (2.5 × 10^4^ cells/well) were seeded onto 96-well plates, and stimulated the next day as described above. After being incubated for the indicated times, MTT (0.5 mg/ml) was added and the cells were then incubated for a further 4 hours. The medium was then removed and cells were dissolved in DMSO (100 μl/well). The OD_570_ was measured using the iMark Microplate Absorbance Reader (Bio-Rad, Hercules, CA).

### Quantitative real-time PCR

RAW264 cells (1.25 × 10^5^ cells/well) were seeded onto 24-well plates and cells were stimulated the next day. After being incubated for the indicated times, total RNA was extracted with RNAiso Plus (Takara, Shiga, Japan) according to the manufacturer’s instructions. Total RNA was reverse-transcribed using the PrimeScript RT reagent kit (Takara) and then PCR-amplified by Takara PCR Thermal Cycler Dice (Takara) using SYBR Premix Ex Taq II (Takara). The following oligonucleotides were used for PCR: 18SrRNA: (forward) 5′-TTGACGGAAGGGCACCACCAG-3′ and (reverse) 5′ GCACCACCACCCACGGAATCG-3′, GAPDH: (forward) 5′- TGTGTCCGTCGTGGATCTGA-3′ and (reverse) 5′-TTGCTGTTGAAGTCGCAGGAG-3′, IL-6: (forward) 5′-AGTTGCCTTCTTGGGACTGA-3′ and (reverse) 5′-CAGAATTGCCATTGCACAAC-3′, TNF-α: (forward) 5′-CCTCCCTCTCATCAGTTCTA-3′ and (reverse) 5′-ACTTGGTGGTTTGCTACGAC-3′, IL-1β: (forward) 5′-GAAGAAGAGCCCATCCTCTG-3′ and (reverse) 5′- TCATCTCGGAGCCTGTAGTG-3′, inducible nitric-oxide synthase (iNOS): (forward) 5′-GGAGCGAGTTGTGGATTGTC-3′ and (reverse) 5′-GTGAGGGCTTGGCTGAGTGAG-3′, IL-10: (forward) 5′-AGCCGGGAAGACAATAACTG-3′ and (reverse) 5′-CATTTCCGATAAGGCTTGG-3′, COX-2: (forward) 5′-GAAGTCTTTGGTCTGGTGCCTG-3′ and (reverse) 5′-GTCTGCTGGTTTGGAATAGTTGC-3′, MCP-1: (forward) 5′-CCTGTCATGCTTCTGGGCCTGC-3′ and (reverse) 5′-GGGGCGTTAACTGCATCTGGCTG-3′, IL-12B: (forward) 5′-TGGAAGCACGGCAGCAGAATAAAT-3′ and (reverse) 5′-TGCGCTGGATTCGAACAAAGAACT-3′, and Arid5a: (forward) 5′-CTGTCCTACGCAACAGACTGG-3′ and (reverse) 5′-GAAGTGAGGTGCCGCATAGG-3′. Normalization and fold changes were calculated using the ΔΔCt method.

### Luciferase assay

RAW264 cells (7.5 × 10^4^ cells/well) were seeded onto 24-well plates and, the next day, cells were transfected with the IL-6 promoter reporter plasmid (500 ng/well) and control luciferase reporter plasmid (160 ng/well) in 48 μl of Opti-MEM (Invitrogen) per well using the X-tremeGENE HP DNA transfection reagent (Roche, Basel, Switzerland) according to the manufacturer’s instructions. Cells were washed two times with phosphate-buffered saline (PBS) after 24 hours, and stimulated with LPS in the absence or presence of NiCl_2_. Cell lysates were prepared 8 hours later and luciferase activity was determined using a dual-luciferase reporter assay system (Promega).

### Immunoblotting

RAW264 cells (2.5 × 10^5^ cells/well) were seeded onto 12-well plates and the cells were stimulated the next day as described above. After the stimulation for the indicated times, cells were washed two times with ice-cold PBS and lysed in ice-cold lysis buffer (20 mM HEPES, pH 7.4, 1% (v/v) Triton-X 100, 10% (v/v) glycerol, 50 mM sodium fluoride, 2.5 mM *p*-nitrophenyl phosphate, 10 μg/ml phenylmethylsulfonyl fluoride, 1 mM Na_3_VO_4_, 10 μg/ml leupeptin, and 1 mM EDTA). The proteins in the cell lysates were separated using SDS—PAGE and transferred electrophoretically onto a nitrocellulose membrane (GE Healthcare, Buckinghamshire, England). Regnase-1, actin, Arid5a, p44/42 MAPK (ERK1/2), p38 MAPK, and JNK were detected by immunoblotting using a rabbit anti-regnase-1 antibody [28], goat anti-actin antibody (Santa Cruz Biotechnology Inc., Santa Cruz, CA), rabbit anti-Arid5a antibody (Abcam, Cambridge, USA), rabbit anti-ERK1/2 antibody (Upstate, Biotechnology, Lake Placid, NY), rabbit anti-p38 MAPK antibody (Santa Cruz), and rabbit anti-SAPK/JNK antibody (Cell Signaling Technology, Beverly, MA), respectively. The phosphorylation of ERK1/2, p38 MAPK, and JNK was detected using a rabbit anti-phospho-ERK1/2 (Thr202/Tyr204) antibody (Cell Signaling), rabbit anti-phospho-p38 MAPK (Thr180/Tyr182) antibody (Cell Signaling), and rabbit anti-phospho-JNK antibody (Promega), respectively. Proteins were detected using a chemiluminescence detection system (ECL system, PerkinElmer Life Sciences, Boston, MA).

### Statistical analysis

Values in the figures are expressed as means from the indicated number of samples with S.E.M. shown by vertical bars. The significance of the results was analyzed using a one-way ANOVA with Dunnett’s post-hoc test.

## Results

### NiCl_2_ inhibited the LPS-induced production of IL-6, but not that of TNF-α in the air pouch-type inflammation model

**Fig 1:**
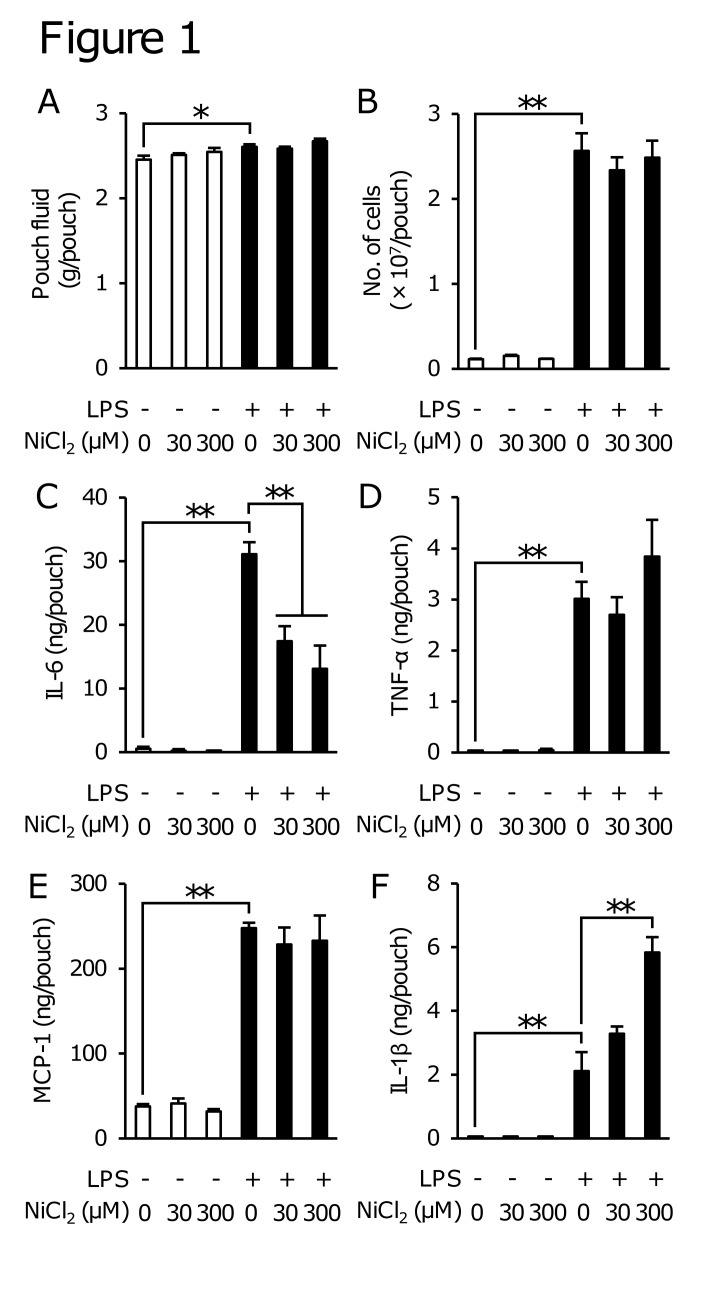
Effects of NiCl2 on LPS-induced inflammation in mice. Two milliliters of CMC-Na solution containing NiCl_2_ (30 or 300 μM) in the presence or absence of LPS (10 ng/ml) was injected into the air pouch, and the pouch fluid was then collected 8 h after the stimulation. The pouch fluid weight (A) and number of leukocytes in the pouch fluid (B) were measured and the concentrations of IL-6 (C), TNF-α (D), MCP-1 (E), and IL-1β (F) were determined by ELISA. Data represent the mean ± S.E.M. (n = 6, 7). *p < 0.05 and **p < 0.01 between the indicated groups.

To investigate whether Ni^2+^ modulates LPS-induced inflammation *in vivo*, we evaluated the effects of NiCl_2_ on the infiltration of leukocytes and production of cytokines using an air pouch-type LPS-induced inflammation model. In this model, LPS slightly increased the weight of pouch fluid collected 8 h after the injection, and appeared to increase the number of leukocytes and the levels of IL-6, TNF-α, IL-1β, and MCP-1 in the pouch fluid (Fig. 1). On the other hand, NiCl_2_ (30 and 300 μM) itself almost did not induce these inflammatory responses (Fig. 1A and B). NiCl_2_ did not affect the LPS-induced production of TNF-α and MCP-1, but enhanced that of IL-1β. Unexpectedly, NiCl_2_ inhibited the LPS-induced increases in IL-6 levels in the pouch fluid (Fig. 1C-F).

### NiCl_2_ and CoCl_2_ inhibited the LPS-induced production of IL-6, but not that of TNF-α in RAW264 cells

**Fig 2:**
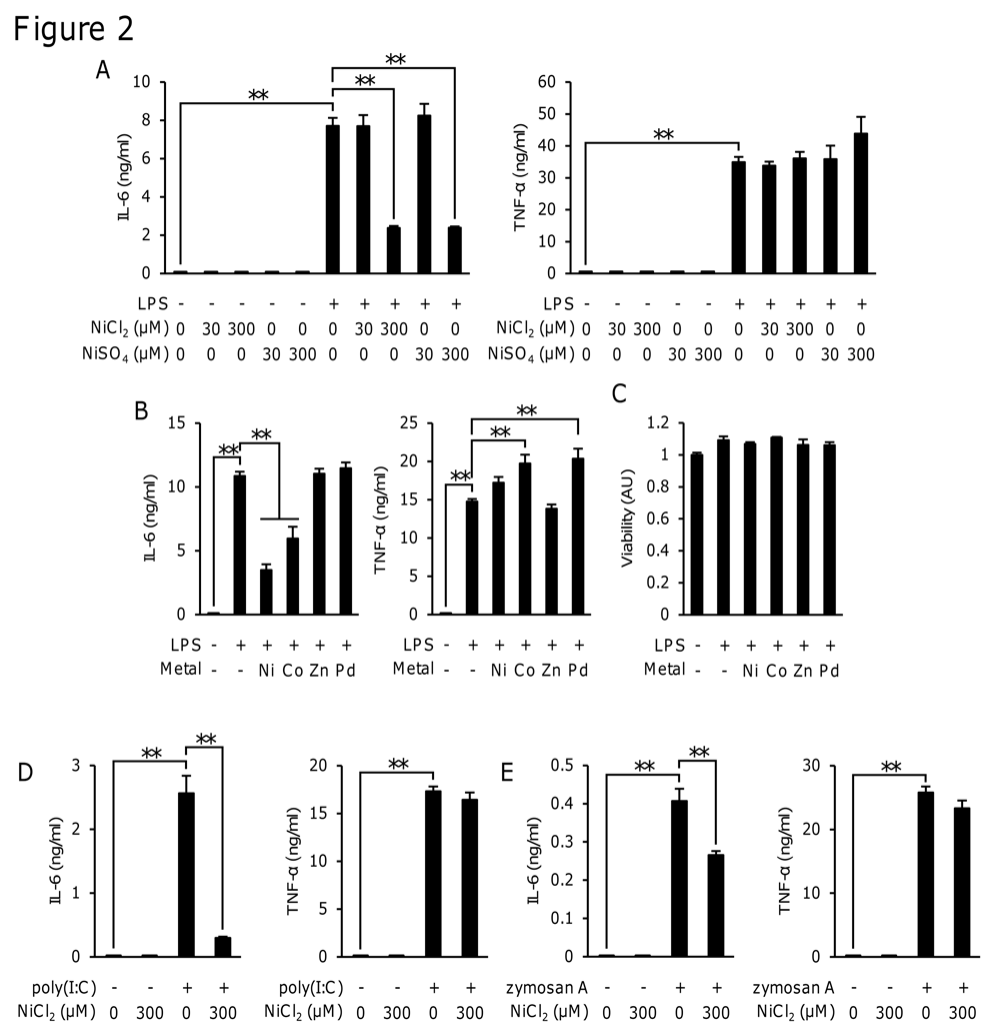
Effects of various metal ions on the LPS-induced production of IL-6 and TNF-α in RAW264 cells. RAW264 cells were incubated for 8 h in medium containing LPS (1 μg/ml) (A, B, and C), poly(I:C) (50 μg/ml) (D), or zymosan A (500 μg/ml) (E) in the presence or absence of NiCl_2_ or NiSO_4_ at the indicated concentrations, or 300 μM of NiCl_2_, CoCl_2_, ZnCl_2_, or PdCl_2_ (B and C). The concentrations of IL-6 and TNF-α in the medium were determined by ELISA and cell viability was determined by the MTT assay. The mean value of the control was set to 1.0 (C). Data represent the mean ± S.E.M. (*n* = 3, 4). **p < 0.01 between the indicated groups. Data are representative of three independent experiments.

RAW264, a murine macrophage cell line, was used to analyze the mechanism responsible for inhibiting the LPS-induced production of IL-6 by NiCl_2_. We first examined which ions exerted the inhibitory effects. The stimulation of RAW264 cells with LPS (1 μg/ml) for 8 h induced the production of IL-6 and TNF-α. Both NiCl_2_ (300 μM) and NiSO_4_ (300 μM) inhibited the LPS-induced production of IL-6, but not that of TNF-α (Fig. 2A). CoCl_2_ (300 μM), but not ZnCl_2_ (300 μM) or PdCl_2_ (300 μM) also inhibited this production of IL-6, whereas none of these inhibited the production of TNF-α (Fig. 2B). None of the metal ions or LPS affected cell viability (Fig. 2C). These results suggested that this inhibitory action was a common feature between Ni^2+^ and Co^2+^. The TLR3 ligand poly(I:C) (50 μg/ml) and TLR2 ligand zymosan A (500 μg/ml) also induced the production of both IL-6 and TNF-α. NiCl_2_ inhibited the poly(I:C)- and zymosan A-induced production of IL-6, but not that of TNF-α (Fig. 2D and E).

### NiCl_2_ inhibited the expression of IL-6 only among the LPS-induced inflammatory proteins

**Fig 3:**
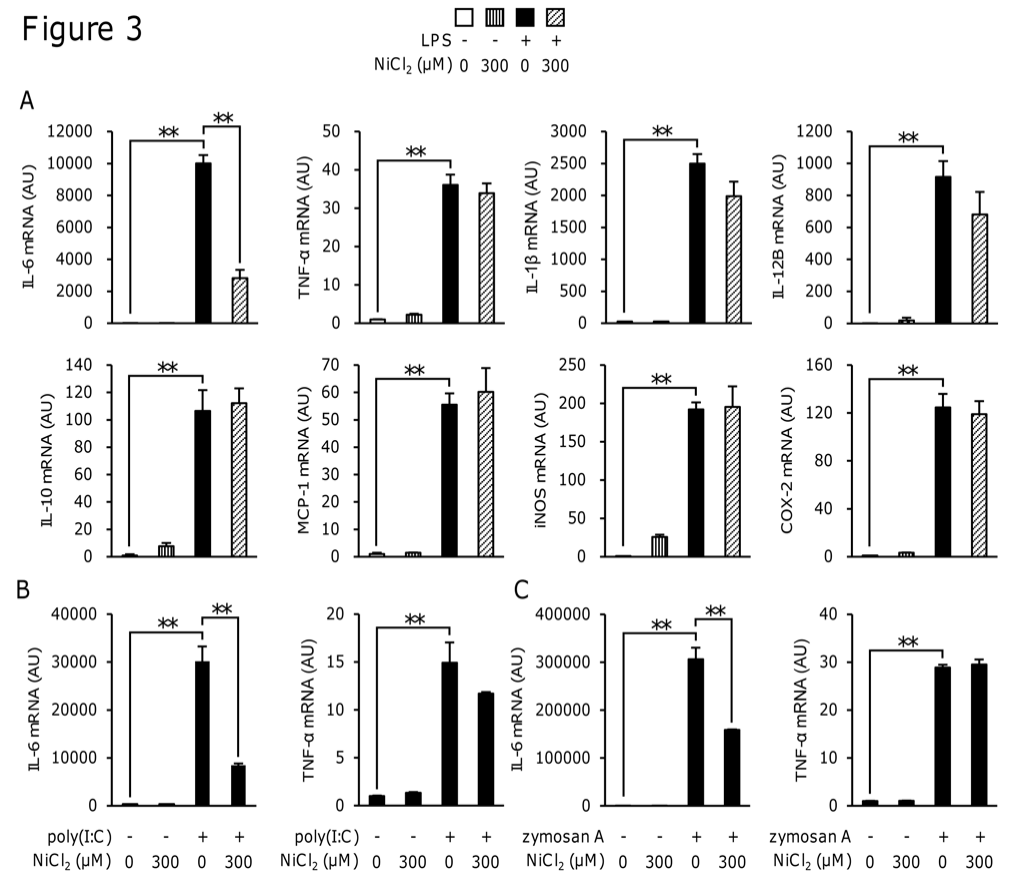
Effects of NiCl2 on the LPS-induced expression of mRNA for inflammatory proteins. RAW264 cells were incubated for 4 h in medium containing LPS (1 μg/ml) (A), poly(I:C) (50 μg/ml) (B), or zymosan A (500 μg/ml) (C) in the presence or absence of NiCl_2_ (300 μM), and mRNAs for various inflammatory proteins were then measured by quantitative real-time PCR. Values are normalized to those of 18 S rRNA and the mean value of the control was set to 1.0. Data represent the mean ± S.E.M. (*n* = 4). **p < 0.01 between the indicated groups. Data are representative of two independent experiments.

To determine whether NiCl_2_ inhibited other LPS-induced inflammatory proteins, we investigated the effects of NiCl_2_ on the LPS-induced expression of the mRNA of various inflammatory proteins in RAW264 cells. The stimulation of RAW264 cells with LPS for 4 h induced the expression of IL-6, TNF-α, IL-1β, IL-12B, MCP-1, iNOS, COX-2, and IL-10 mRNA. Among these cytokines, NiCl_2_ only inhibited the expression of IL-6 mRNA (Fig. 3A). NiCl_2_ also inhibited the poly(I:C)- and zymosan A-induced expression of mRNA of IL-6, but not that of TNF-α (Fig. 3B and C).

### NiCl_2_ did not inhibit the LPS-induced phosphorylation of ERK1/2, p38 MAPK, or JNK

**Fig 4:**
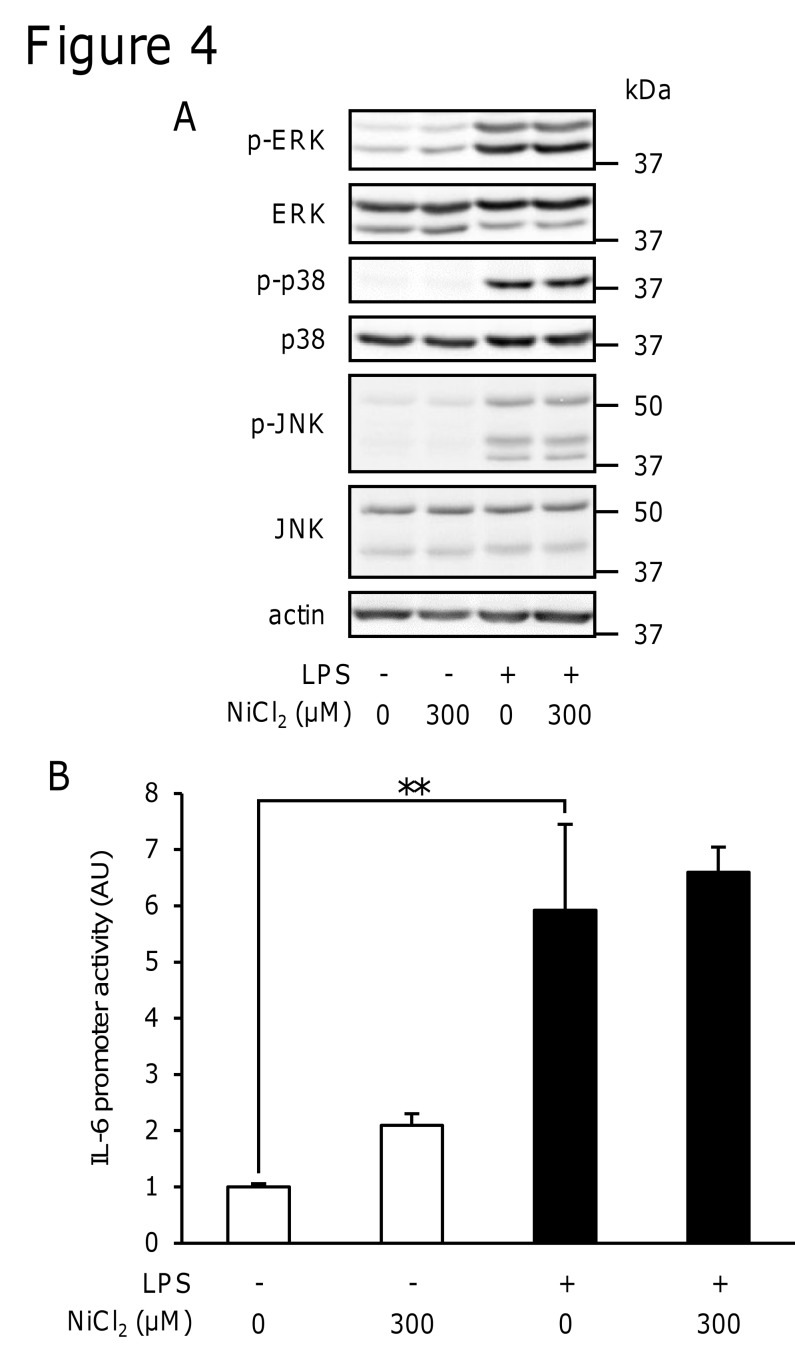
Effects of NiCl2 on the LPS-induced phosphorylation of ERK1/2, p38 MAPK, and JNK as well as IL-6 promoter activity. (A) RAW264 cells were incubated in medium containing LPS (1 μg/ml) in the presence or absence of NiCl_2_ (300 μM) for 15 min. The protein levels of p-ERK, ERK, p-p38, p38, p-JNK, JNK, and actin were determined in cell lysates by immunoblotting. Data are representative of three independent experiments. (B) RAW264 cells were transiently transfected with a luciferase gene construct with the IL-6 promoter and, 24 h later, were then stimulated for 8 h with LPS (1 μg/ml) in the presence or absence of NiCl_2_ (300 μM). Luciferase activity was determined in these cells. Values are normalized to those of *Renilla* luciferase activity and the mean value of the control was set to 1.0. Data represent the mean ± S.E.M. (*n* = 3). **p < 0.01 between the indicated groups. Data are representative of two independent experiments.

The production of various inflammatory proteins including IL-6 and TNF-α is regulated by MAPK signaling. We examined the phosphorylation of ERK1/2, p38 MAPK, and JNK using immunoblotting to investigate the effects of NiCl_2_ on LPS-induced signal transduction. LPS induced the phosphorylation of ERK1/2, p38 MAPK, and JNK at 15 min. However, NiCl_2_ did not inhibit their phosphorylation (Fig. 4A).

### NiCl_2_ did not inhibit IL-6 promoter activity

We examined the effects of NiCl_2_ on IL-6 promoter activity. Although NiCl_2_ reduced IL-6 mRNA levels (Fig. 3), it did not inhibit LPS-induced IL-6 promoter activity (Fig. 4B). In the same experiment, NiCl_2_ was confirmed to inhibit the LPS-induced production of IL-6, but not that of TNF-α in RAW264 cells transfected with plasmids (data not shown). These results suggested that NiCl_2_ decreased IL-6 mRNA stability rather than inhibiting its transcription.

### NiCl_2_ promoted IL-6 mRNA degradation

**Fig 5:**
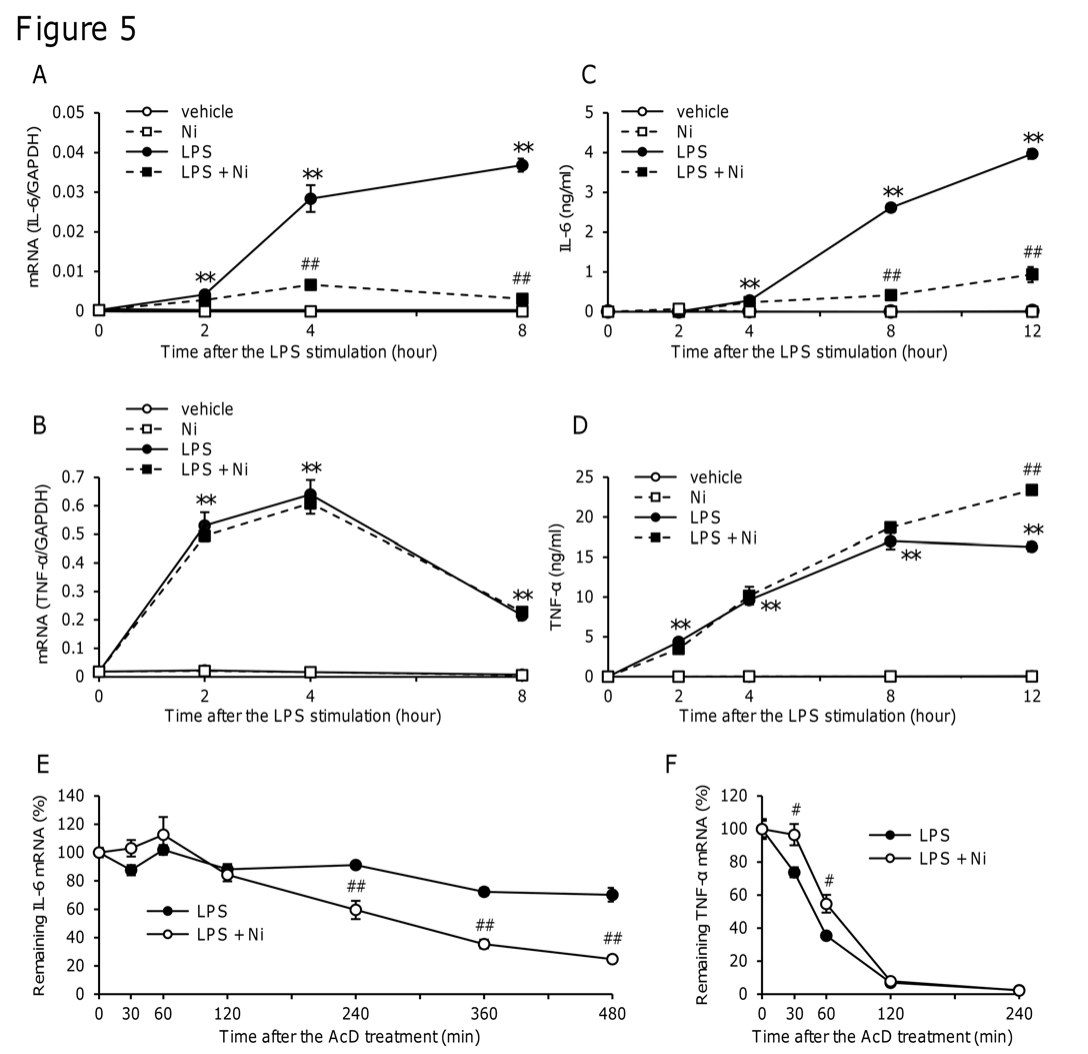
Time course of IL-6 and TNF-α expression and effects of NiCl2 on the stability of IL-6 and TNF-α mRNA. RAW264 cells were incubated in medium containing LPS (1 μg/ml) in the presence or absence of NiCl_2_ (300 μM) for the indicated time. (A and B) IL-6 (A) and TNF-α mRNA (B) levels were measured by quantitative real-time PCR. Values are normalized to those of GAPDH and the mean value of the control was set to 1.0. (C and D) IL-6 (C) and TNF-α (D) concentrations were determined by ELISA. Data represent the mean ± S.E.M. (n = 3, 4). (E and F) RAW264 cells were incubated in medium containing LPS (1 μg/ml) in the presence or absence of NiCl_2_ (300 μM). After the 4 h stimulation, AcD (5 μg/ml) was added and the cells were further incubated for the indicated time. IL-6 (E) and TNF-α (F) mRNA levels were determined by quantitative real-time PCR. Values are normalized to those of GAPDH and the mean value of the control at time 0 was set to 1.0. Data represent the mean ± S.E.M. (*n* = 4). **p < 0.01 vs. vehicle, *#p* < 0.05 and *##p* < 0.01 vs. LPS. Data are representative of two independent experiments.

To clarify when IL-6 and TNF-α mRNA levels reached a maximum in LPS-stimulated cells, time changes in the LPS-induced production of IL-6 and TNF-α and effects of NiCl_2_ were examined. IL-6 mRNA levels increased from 4 h after the stimulation and were further, but slightly increased after 8 h (Fig. 5A). In contrast, the LPS-induced increase in TNF-α mRNA reached a maximum after 2 h and then decreased rapidly (Fig. 5B). NiCl_2_ inhibited the LPS-induced production of IL-6 mRNA after 4 and 8 h (Fig. 5A) and protein after 8 and 12 h (Fig. 5C), but not those of TNF-α (Fig. 5B and D). Based on these time changes, AcD, a transcription inhibitor, was added 4 h after the stimulation to stop transcription, and decreases in mRNA levels were thereafter examined as an index of the stability of mRNA. The decrease in IL-6 mRNA levels was slower than that in TNF-α mRNA after the addition of AcD (Fig. 5E and F). NiCl_2_ significantly decreased the LPS-induced expression of IL-6 mRNA 240 min after the AcD treatment (Fig. 5E), but slightly increased TNF-α mRNA expression (Fig. 5F). These results indicated that NiCl_2_ selectively decreased the stability of IL-6 mRNA.

### NiCl_2_ did not affect regnase-1 expression

**Fig 6:**
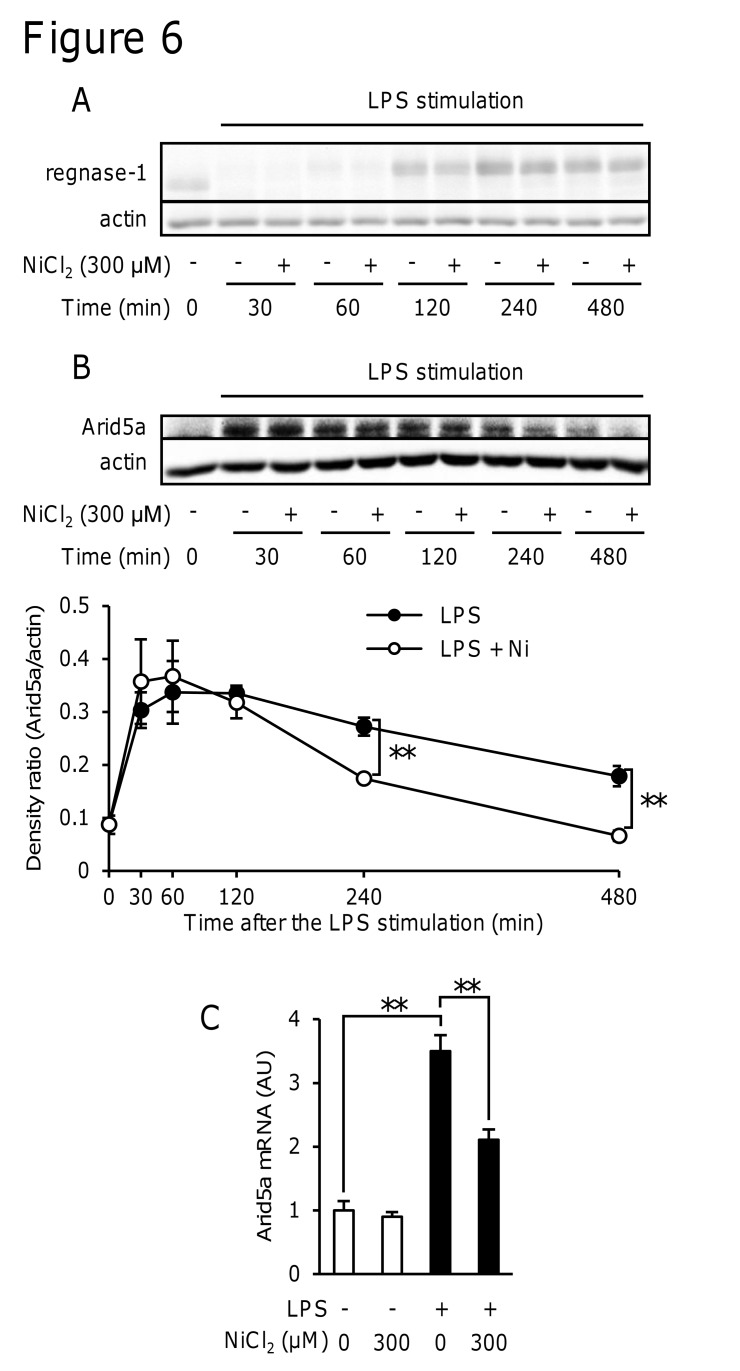
Effects of NiCl2 on LPS-induced changes in regnase-1 and Arid5a expression levels. RAW264 cells were incubated in medium containing LPS (1 μg/ml) in the presence or absence of NiCl_2_ (300 μM) for the indicated periods (A and B) and 4 h (C). (A and B) Regnase-1, actin, and Arid5a protein levels in cell lysates were determined by immunoblotting. Protein levels of Arid5a and actin were quantified using ImageJ software (B). Data represent the mean ± S.E.M. (n = 4). Data are representative of three independent experiments. (D) Arid5a mRNA levels were measured by quantitative real-time PCR. Values are normalized to those of GAPDH and the mean value of the control was set to 1.0. Data represent the mean ± S.E.M. (*n* = 4). **p < 0.01 between the indicated groups. Data are representative of two independent experiments.

Since regnase-1 is an RNase that selectively degrades IL-6 mRNA, we first examined whether NiCl_2_ affected the expression of regnase-1. When RAW264 cells were stimulated with LPS, the expression of regnase-1 decreased 30–60 min after the LPS stimulation and then recovered after 240–480 min (Fig. 6A). NiCl_2_ did not affect LPS-induced time changes in the expression of regnase-1 (Fig. 6A).

### NiCl_2_ inhibited the LPS-induced expression of Arid5a mRNA and protein levels

We next examined the effects of NiCl_2_ on the LPS-induced expression of Arid5a, a stabilizer of IL-6 mRNA. The expression of the Arid5a protein was induced by the stimulation with LPS, while NiCl_2_ decreased the protein level at 4 h and 8 h (Fig. 6B). NiCl_2_ also inhibited LPS-induced Arid5a mRNA expression 4 h after the stimulation (Fig. 6C).

## Discussion

In the present study, we demonstrated that Ni^2+^ selectively inhibited the LPS-induced production of IL-6 both *in vivo* and *in vitro*. The results obtained also indicated that Ni^2+^ decreased the LPS-induced expression of Arid5a, and, as a result, reduced the stability of IL-6 mRNA. This is the first study to demonstrate that Ni^2+^ post-transcriptionally inhibited the expression of cytokines, and also provides a new insight into Ni^2+^-induced cellular events.

The activation of Toll-like receptor 4 (TLR4) by LPS is known to initiate signaling events and induce the secretion of inflammatory cytokines, chemokines, type I IFN, and antimicrobial peptides [29]. These proteins evoke inflammation by activating neutrophils, macrophages, and dendritic cells. The LPS stimulation has also been shown to induce the production of PGE_2_, IL-1β, IL-6, TNF-α, and other cytokines in the air pouch-type inflammation model [27, 30]. We showed that NiCl_2_ inhibited the LPS-induced production of IL-6 in this model, but did not affect the infiltration of leukocytes or production of TNF-α and MCP-1 (Fig. 1A-E). The enhancement by NiCl_2_ of the LPS-induced production of IL-1β in this model (Fig. 1F) may be attributed to the activation of inflammasomes because Ni^2+^ has been shown to activate inflammasomes and promote the release of IL-1β [25]. These results indicated that the inhibitory action of NiCl_2_ was highly selective to the production of IL-6.

To clarify the molecular mechanisms by which Ni^2+^ inhibited the LPS-induced production of IL-6, we analyzed the effects of Ni^2+^ on the production of cytokines in RAW264 cells, a murine macrophage-like cell line. The results obtained in RAW264 cells also showed that NiCl_2_ selectively inhibited the production of IL-6 (Figs. 2A and 3A). Of the metal ions tested, the inhibitory effects on IL-6 were limited to Ni^2+^ and Co^2+^ (Fig. 2B). Ni^2+^ and Co^2+^ are known to have similar effects on signaling molecules. Previous studies showed that both Ni^2+^ and Co^2+^ induced the expression of HIF-1α [31], bound to the histidine residues of peptides/proteins [32], and activated human TLR4 but not mouse TLR4 [33]. The result that Ni^2+^ inhibited the poly(I:C)- or zymosan A-induced production of IL-6, but not that of TNF-α (Fig. 2D and E) indicated that these effects were not specific to TLR4-mediated responses. This is the first study to show that Ni^2+^ and Co^2+^ commonly inhibited the production of IL-6 and also suggests a novel common target of Ni^2+^ and Co^2+^.

LPS is known to activate TLR4 and signaling molecules such as ERK1/2, p38 MAPK, JNK, and NF-κB, which induce the expression of inflammatory proteins [34]. Our results suggested that Ni^2+^ inhibited unidentified pathways, except for the activation of MAP kinases, such as ERK1/2, p38 MAPK, and JNK (Fig. 4A). In addition, Ni^2+^ did not inhibit transcriptional activity in the reporter gene assay using the IL-6 promoter region, which includes-1232 to +39. Since the promoter region contains the binding sites of the main transcription factors that regulate the expression of IL-6, such as NF-κB, AP-1, NF-IL6, and CREB [23, 26], it is likely that Ni^2+^ did not inhibit the activation of these transcription factors (Fig. 4B). We instead found that Ni^2+^ decreased the stability of mRNA for IL-6, but not TNF-α (Fig. 5E and F).

Some proteins are involved in the post-transcriptional regulation of cytokine mRNAs. HuR and the activation of p38 MAPK have been shown to stabilize some cytokine mRNAs [35, 36, 37], whereas tristetraprolin (TTP) promotes the degradation of some cytokine mRNAs [38]. However, these proteins did not exhibit specificity to IL-6 mRNA. Regnase-1 [24] and Arid5a [25] have recently been identified as specific regulators of the degradation of IL-6 mRNA. Regnase-1 is a cytoplasmic protein that is composed of a PIN-like RNase domain and CCCH-type zinc-finger domain [24]. Regnase-1 was previously shown to bind to the 3′-UTR of IL-6 mRNA and degrade IL-6 mRNA [28]. Arid5a is one of the ARID (AT-rich interactive domain) family proteins that play important roles in development, tissue-specific gene expression, and proliferation [39]. Arid5a was shown to bind to and stabilize IL-6 mRNA [25]. Since the activities of both regnase-1 and Arid5a were affected at the protein levels, we analyzed the effects of Ni^2+^ on the expression of regnase-1 and Arid5a. We found that NiCl_2_ did not affect LPS-induced changes in regnase-1 (Fig. 6A), but inhibited the LPS-induced increase in Arid5a (Fig. 6B and C). These results indicate that Ni^2+^ destabilized IL-6 mRNA by inhibiting the induction of Arid5a. A previous study reported that when the expression of Arid5a was decreased by siRNA, the LPS-induced production of IL-6 was also decreased, whereas that of TNF-α remained unchanged [25].

Ni^2+^ has been shown to induce the expression of HIF-1α and this is mediated by the inhibition of prolyl hydroxylase and HIF-2α expression [31, 40, 41]. Activated HIF-1α and-2α were previously reported to enhance inflammatory proteins such as COX-2, iNOS, IL-1β, IL-6, IL-12, and TNF-α [31, 40, 41]. However, the target protein of Ni^2+^ involved in the inhibition of IL-6 production has yet to be identified. Although LPS, IL-1β, and IL-6 are known to induce the expression of Arid5a [25], the molecular mechanisms regulating the expression of Arid5a remain unknown. Ni^2+^ and nickel complexes have been shown to inhibit the activation of NF-κB. For example, a nickel complex inhibited the LPS-induced expression of IL-6 and TNF-α mRNA through docking to the kinase domain of IKKβ [42]. Ni^2+^ also binds to the p50 subunit of NF-κB and inhibit its accumulation in the nucleus as well as the expression of IL-8 in humans [43]. However, it is unlikely that Ni^2+^ inhibited the production of Arid5a by inhibiting NF-κB as described above. Ni^2+^ can interact with many proteins at the histidine residue and this may affect their functions. Some Ni^2+^-binding proteins may be target molecules that mediate the Ni^2+^-induced inhibition of Arid5a expression. A proteomic analysis using Ni-NTA beads has been used to identify Ni^2+^-binding proteins [44]. The target protein of Ni^2+^ is currently being investigated using this method.

The prevalence of metal allergies in patients with a metal implant is higher than that in the general population [9]. Implant failure causes the corrosion of metals and elution of metal ions such as Ni^2+^. The Ni^2+^-induced activation of TLR4 is an important factor in human Ni allergies [45]. Therefore, in humans, Ni^2+^ may have opposite effects on the production of IL-6 via the stimulation of TLR4 and selective inhibition of IL-6. In Ni allergy model mice, LPS promotes sensitization to Ni^2+^ [12] and augments the allergic responses induced by Ni^2+^ [13]. Ni^2+^ forms a complex with some proteins and activates naïve T cells [9]. Previous studies reported that Ni allergies are type IV allergies that are mediated by Th1 cells [46]. Th17 cytokines such as IL-17 also play a role in Ni allergies [47]. IFN-γ- and IL-17-producing T cells, but not pure Th17 cells mainly produce IL-17 in Ni allergy patients [48]. IL-17 could amplify T cell-mediated skin immune responses by enhancing the IFN-γ-induced expression of ICAM-1 [47]. Thus, in metal allergies, the immune balance is shifted to Th1/Th17 responses. However, it currently remains unclear how Ni^2+^ affects the immune balance and enhances Th17 responses. T cell-producing cytokines such as IFN-γ and IL-17 were not detected in the air pouch model or RAW264 cells. IL-6 acts on T cells and induces Th2 responses [19], resulting in the inhibition of Th1 responses [20]. IL-6 has also been shown to augment TGF-β-induced Th17 differentiation [22]. Therefore, Ni^2+^ may affect T cell differentiation by inhibiting the production of IL-6. Using a Ni allergy model and implant model, further studies to determine whether Ni^2+^ induces a shift in the cytokine balance by inhibiting the production of IL-6 are required.
